# Supplementary material for: Rate of brain aging and APOE ε4 are synergistic risk factors for Alzheimer’s disease
Source: Life Sci Alliance. 2019 May 27;2(3):e201900303. doi: 10.26508/lsa.201900303 (PMC6537750; doi:10.26508/lsa.201900303)
Supplement: Supplementary file 3 [file LSA-2019-00303_TableS3.docx]

Table S3

| ROS-MAP Cohort Characteristics | n=438 |
| --- | --- |
| Age range | 67-108 years |
| Sex female | 275 subjects |
| Average education | 16.5 years |
| Race white | 437 subjects |
| Race black | 1 subject |
| Race Spanish | 3 subjects |
| Average post mortem interval | 7.1 hrs |
| Past medical history of AD | 170 subjects |
| Pathological diagnosis of AD | 261 subjects |
| Past medical history of PD | 31 subjects |
| Past medical history of Lewy body dementia | 11 subjects |
| Self report history of thyroid problems | 73 subjects |
| Self report history of heart problems | 72 subjects |
| Self report history of stroke | 43 subjects |
| Self report history of cancer | 143 subjects |
| Self report history of hypertension | 203 subjects |
