# Supplementary material for: Rate of brain aging and APOE ε4 are synergistic risk factors for Alzheimer’s disease
Source: Life Sci Alliance. 2019 May 27;2(3):e201900303. doi: 10.26508/lsa.201900303 (PMC6537750; doi:10.26508/lsa.201900303)
Supplement: Supplementary file 4 [file LSA-2019-00303_TableS4.docx]

Table S4

|  | ΔAge | |
| --- | --- | --- |
|  | Control | Disease |
| ΔGlobal cognition/yr | 0.009 | 2.5E-04 |
| ΔEpisodic memory/yr | 0.009 | 0.0035 |
| ΔVisual-spatial ability/yr | 0.2 | 0.003 |
| ΔPerceptual speed/yr | 0.01 | 0.0012 |
| ΔSemantic memory/yr | 0.08 | 3.2E-04 |
| ΔWorking memory/yr | 0.5 | 0.08 |
| Global cognition level | 0.001 | 1.9E-04 |
| Episodic memory level | 0.003 | 9.5E-02 |
| AD clinical diagnosis | N/A | N/A |
| Mini Mental Exam score | 0.05 | 0.007 |
