# Supplementary material for: Rate of brain aging and APOE ε4 are synergistic risk factors for Alzheimer’s disease
Source: Life Sci Alliance. 2019 May 27;2(3):e201900303. doi: 10.26508/lsa.201900303 (PMC6537750; doi:10.26508/lsa.201900303)
Supplement: Supplementary file 5 [file LSA-2019-00303_TableS5.docx]

Table S5

| Variable | *methyl* ΔAge | ΔAge |
| --- | --- | --- |
|  | p value | p value |
| ΔGlobal cognition/yr | 0.78 | 1.8E-05 |
| ΔEpisodic memory/yr | 0.34 | 1.2E-04 |
| ΔVisual-spatial ability/yr | 0.9 | 1.3E-02 |
| ΔPerceptual speed/yr | 0.62 | 5.0E-05 |
| ΔSemantic memory/yr | 0.76 | 1.6E-05 |
| ΔWorking memory/yr | 0.74 | 0.02 |
| Global cognition level | 0.51 | 1.5E-06 |
| Episodic memory level | 0.33 | 9E-06 |
| Dementia grade | 0.006 | 7.6E-05 |
| AD clinical diagnosis | 0.51 | 1.3E-05 |
| Mini Mental Exam score | 0.73 | 2.0E-04 |
| Depression score | 0.03 | 0.19 |
| General pathology | 0.2 | 0.04 |
| Plaque level | 0.69 | 0.30 |
| Tangles level | 0.37 | 1.0E-05 |
| Amyloid level | 0.05 | 0.16 |
| PD diagnosis | 0.54 | 0.81 |
| PD sign score | 0.06 | 0.01 |
| Gait | 0.03 | 0.004 |
| Bradykinesia | 0.05 | 0.27 |
| Rigidity | 0.80 | 0.004 |
| Tremor | 0.51 | 0.51 |
| PD Pathology | 0.001 | 0.47 |
| Lewy body pathology | 0.68 | 0.005 |
| Stroke diagnosis | 0.92 | 0.17 |
| "Heart problem" history | 0.90 | 0.03 |
| Hypertension at baseline | 0.29 | 0.003 |
| Arteriolar sclerosis | 0.38 | 0.37 |
| Cerebral infarction gross | 0.28 | 0.65 |
| Cerebral infarction micro | 0.13 | 0.19 |
| Cancer history | 0.43 | 0.74 |
| Thyroid disease history | 0.64 | 0.04 |
| Smoking (lifetime pack-years) | 0.53 | 0.22 |
| APOε4 alleles | 0.15 | 0.02 |
| Δ Age | 0 | 0.035 |
